# Spatiotemporal analysis of projected impacts of climate change on the major C_3_ and C_4_ crop yield under representative concentration pathway 4.5: Insight from the coasts of Tamil Nadu, South India

**DOI:** 10.1371/journal.pone.0180706

**Published:** 2017-07-28

**Authors:** Ramachandran A, Dhanya Praveen, Jaganathan R, RajaLakshmi D, Palanivelu K

**Affiliations:** 1 Centre for Climate Change and Adaptation Research, Anna University, Guindy, Chennai, Tamil Nadu, India; 2 Tamil Nadu Agricultural University, Coimbatore, Tamil Nadu, India; Louisiana State University College of Agriculture, UNITED STATES

## Abstract

India's dependence on a climate sensitive sector like agriculture makes it highly vulnerable to its impacts. However, agriculture is highly heterogeneous across the country owing to regional disparities in exposure, sensitivity, and adaptive capacity. It is essential to know and quantify the possible impacts of changes in climate on crop yield for successful agricultural management and planning at a local scale. The Hadley Centre Global Environment Model version 2-Earth System (HadGEM-ES) was employed to generate regional climate projections for the study area using the Regional Climate Model (RCM) RegCM4.4. The dynamics in potential impacts at the sub-district level were evaluated using the Representative Concentration Pathway 4.5 (RCPs). The aim of this study was to simulate the crop yield under a plausible change in climate for the coastal areas of South India through the end of this century. The crop simulation model, the Decision Support System for Agrotechnology Transfer (DSSAT) 4.5, was used to understand the plausible impacts on the major crop yields of rice, groundnuts, and sugarcane under the RCP 4.5 trajectory. The findings reveal that under the RCP 4.5 scenario there will be decreases in the major C_3_ and C_4_ crop yields in the study area. This would affect not only the local food security, but the livelihood security as well. This necessitates timely planning to achieve sustainable crop productivity and livelihood security. On the other hand, this situation warrants appropriate adaptations and policy intervention at the sub-district level for achieving sustainable crop productivity in the future.

## Introduction

The majority of the world’s undernourished people live in developing countries. Two-thirds live in just seven countries (Bangladesh, China, the Democratic Republic of the Congo, Ethiopia, India, Indonesia, and Pakistan), with 40 percent living in China and India [1]. The increasing challenge of food and livelihood insecurity largely depend on the rate of yield gain of the major cereal crops in a country like India [2].

Global warming due to a rise in greenhouse gases may exacerbate the negative consequences on crop productivity and food security [3, 4]. According to the 5th Assessment Report (AR5) of the Intergovernmental Panel on Climate Change (IPCC), the global (land and ocean) average temperature has risen 0.85°C (0.65–1.06°C) over the period of 1800–2012 [3]. This trend in global warming is predicted to increase during the 21st century under all the Representative Concentration Pathways (RCPs). The projected ranges of temperature increase are 0.3–1.7°C (RCP 2.6), 1.1–2.6°C (RCP 4.5), 1.4–3.1°C (RCP 6.0), and 2.6–4.8°C (RCP 8.5) for the years 2081–2100, relative to 1986–2005 [3]. Such changes in global mean temperature can radically disturb human society and the natural environment [5]; however, the changes in extreme temperature events, such as heat waves, severe winters, summer storms, hot and cold days, and hot and cold nights, may severely impact the agricultural ecosystems [6].

Despite all technological and cultivar advancements, the weather and the climate are continuing to be the uncontrollable factors affecting crop yield. The yield of crops, particularly in rainfed conditions, depends on the climate to an even larger extent [7]. Research on agriculture in the developing nations has focused on the need to have increased cereal production [8, 9]. Though agriculture in India has undergone major structural changes, it is still the mainstay of the economy with about 49% of the labor force depending on agriculture and the allied sectors for their livelihoods. Agriculture supports the vast majority of the low income, the poor, and the vulnerable people in the country; and climate plays a crucial role, as about 55–60% of the area sown is still rainfed. The share of agriculture and allied activities in the Indian gross domestic product (GDP) was 13.7% during 2012–13 [10].

There are numerous direct and indirect impacts reported on crop production due to the overall impacts of climatic attenuations. The yield of crops, particularly in rainfed conditions, depends on the climate to a larger extent. Global warming may adversely affect the biodiversity and exacerbate the desertification due to an increase in evapotranspiration and a likely decrease in rainfall in dry lands (although it may increase globally) [11]. The direct impacts will have implications for the morphology, the physiology, and the phenology of a plant. Increased temperatures will have profound effects on crop development. Higher temperature exposure beyond the threshold level will eventually affect the quality and the quantity of yields of desirable crops, while weeds infest the areas and pests proliferate [12]. The impacts depend on the relative warming at different growth stages: seed germination, seedling emergence, leaf production, leaf expansion, leaf area duration, flower initiation, flower development, pollination, ripening, maturity, vernalization, and dormancy. Varying precipitation patterns augment the likelihood of short-run crop failures and long-run production declines [13]. The strong negative impacts of warming that limit plant growth, metabolism, and productivity worldwide have been reported by various researchers [14, 15]. Detailed evidence of significant impacts on the biophysical changes and socio-economic stresses have also been noted by many scientists around the world [16–19].

To achieve the target of 4% agricultural growth as envisaged in the Government of India's XI Five Year Plan Period (2007–2012), the enhancement of agriculture productivity is a focus. Indian agricultural production and, consequently, the country’s gross domestic product (GDP) show a strong link with the Indian summer monsoon rainfall [20]. In order to meet the food requirement for the ever increasing population of India, the rice production has to be increased to 162 MT by the year 2015, to 171 MT by 2020, to 178 MT by 2025, and to 185 MT by 2030 [21]. Recently, many scientific articles have highlighted the need for studying the impacts of changing temperatures on the yields of major crops. Kumar and Jain [22] suggested that higher temperatures reduce the yields regardless of rainfall, as noted consistently by other researchers. India falls prey to both these conditions, exacerbated by the fast rate of population growth and the need to feed a billion people. India, with a vast stretch of coastline, is one of the most vulnerable countries in the world regarding the likely impacts of climate change [23]. There is projected to be a threefold expansion of the density of the population in the coastal areas, and 50% of the world’s population will occupy the area within 100 km of the coastal areas [24]. In this context, acquiring an in depth knowledge of the intricate relationship between climate and crop performance is imperative [25].

Increased temperatures will have profound effects on crop development. Higher temperature exposure beyond the threshold level eventually affects the quality and the quantity of the yields of desirable crops, while weeds infest the area and pests proliferate [12]. Varying precipitation patterns augment the likelihood of short-run crop failures and long-run production declines [13]. Although there will be gains in some crops in some regions of the world, especially in higher latitudes, the overall impact of climate change on agriculture are expected to be negative, threatening global food security [26, 27]. Thermal stress during the most sensitive crop developmental stages would hamper the crop photosynthesis and the productivity [28–30].

## Methods

### Profile of the study area

**Fig 1:**
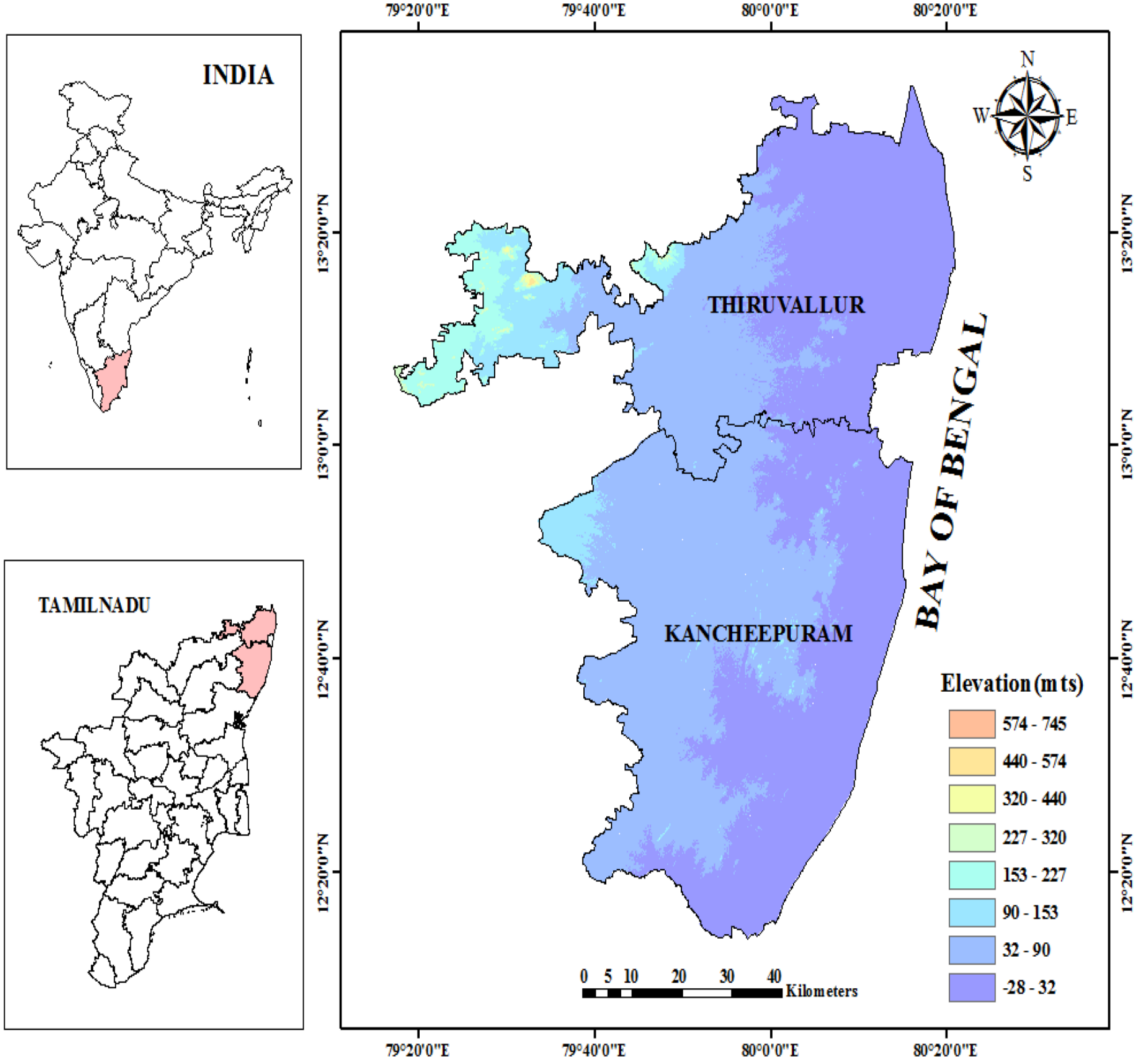
Digital elevation model of the study area.

The former Chengalpet district (presently, the Thiruvallur and Kancheepuram Districts) comes under the northeast Agro Climatic Zone (ACZ) of Tamil Nadu, South India (Fig 1). It lies between the 12° 0’ and 13° 40’N latitudes and 79° 0’ to 80° 20’E longitudes. The majority of its topography is characterized by low lying coastal plains. The geographical setting affects the day and the night time temperatures of the region through the land and the sea breezes. The climate of this region is characterized by a semiarid dry climate in the interior and a semiarid humid climate in the coastal tracks. The general slope of the study area is from the northwest to the southeast. The elevation of this area is below 200 MSL.

The climate of this area is mostly semiarid and dry subhumid with short and intense wet seasons and long dry seasons. The mean day time and night time temperatures of the hottest month (May) are 37°C and 27°C, respectively. The mean day time and the night time temperatures of the coolest month (January) range between 20°C and 29°C. The average rainfall received in this area is 1100 mm/year. As the study area shares the coastal boundaries of the Bay of Bengal, it is highly sensitive to future potential climate change. Owing to the frequent attacks of tropical cyclones and storm surges, agriculture in these coastal tracks is severely impacted.

Crop production plays a major role in the economy of the study area. Besides providing livelihood, it plays an inevitable role in the pattern of food consumption and local food security. The large scale expansion of industries and the intrusions of real estate in the farm lands have reduced the cultivable area.

In this study, extensive field work has been carried out to understand the crop production strategies of the former Chengalpet district, which is now bifurcated as the Thiruvallur and the Kancheepuram districts. The primary and the secondary data on the crop area coverage, the crop production, and the crop yield were collected from the Department of Agriculture Economics and Statistics, Chennai. Literature reviews featuring the seasonal crop reports were also completed.

### Review of crop production practices by fieldwork

Continuous field observations have been carried out to understand the real time situation of the agronomical practices and the management for the major crops rice, groundnut, and sugarcane. Details of the crop production, the phenological details of the major varieties, and the experimental trial outcomes were observed and gathered with the constant support of various scientists working at the Agricultural Research Stations under the control of the Tamil Nadu Agricultural University, namely, the Rice Research Station-Tamil Nadu Agricultural University Tirur, Thiruvallur; the Groundnut Research Station, Tindivanam; and the Sugarcane Research and Breeding Centre, Pondicherry.

### Crop production practices

As far as rice crop cultivation periods are concerned, the farmers cultivate short, medium, and long duration varieties in various seasons. There are unique names locally for each season and for crops like Kharif and Rabi in the Indian crop production scenario. The main seasons in the study area were the Sambha, which is dependent on the northeast monsoon (i.e., the post monsoon rainfall) and the Kuruvai cultivation, which relies on the southwest monsoon showers. The rice crop calendar starts with Sambha (Aug-November), and is followed by Navarai (Dec-Jan) and Sornavari (April to July), which are purely dependent on the tube well and the tank irrigation. The major rice varieties grown in each of these seasons are ADT 37, 47, Mdu-5, and ADT^®^-45. They are also known as Sornavari and Navarai, as they are short duration varieties. These main varieties take 105 to 110 days for maturity. In contrast, during Sambha, medium- and long-duration varieties are grown, including IWP, ADT44, ADT-43, CR1009, ADT38, TRY1, 2. These have a 135–150 day maturity time.

With respect to the groundnut crop production, the major cultivating season is found to be the winter, locally known as Margazhi Pattam (December-January). The main varieties grown during this time are TMV 7, VRI-2, ALR3, and CO(gn). During Masi Pattam (February-March), the TMV7, CO-2, and VRI-2 varieties are cultivated; and during summer or Chithirai Pattam (April-May), CO-2, CO-3, and VRI-2 varieties are cultivated.

As far as sugarcane cultivation practices are concerned, there are three different seasons. The primary season is between December to January, with the varieties of CO658, CO62174, and CO62198 being cultivated; (ii) the midseason, February to March, with COC-85019, COC99061, and CO-86032 being cultivated; and (iii) the end season, April to May, when varieties such as COC-93076, COC85019, and COC99061 are grown.

An overview of the latest crop production strategies in the district indicated the introduction of a System of Rice Intensification (SRI) practices. The farmers are found to be practicing crop residue burning after every rice harvest to obtain increased production. An introduction of the System of Sugarcane Intensification (SSI) practices was also seen in some parts of the study area.

It is to confirm that the present field study did not involve in any of the endangered or protected species.

### Projecting future climate change scenarios

The projections are considered as the preliminary step forward in any climate change impact assessment [31, 32]. The downscaled output has been collected from the Agro Climate Research Center, Tamil Nadu Agricultural University, Coimbatore (TNAU), India. Fine resolution (25 km) climate data that are downscaled using the regional climate model, RegCM-Had Gem2-ES, were employed in this research. Studies based on the latest emission trajectories (i.e., Representative Concentration Pathways (RCPs)) are very limited on impact assessment, especially for the Indian scenarios, which are in the infancy stage.

#### RegCM -regional climate model

**Fig 2:**
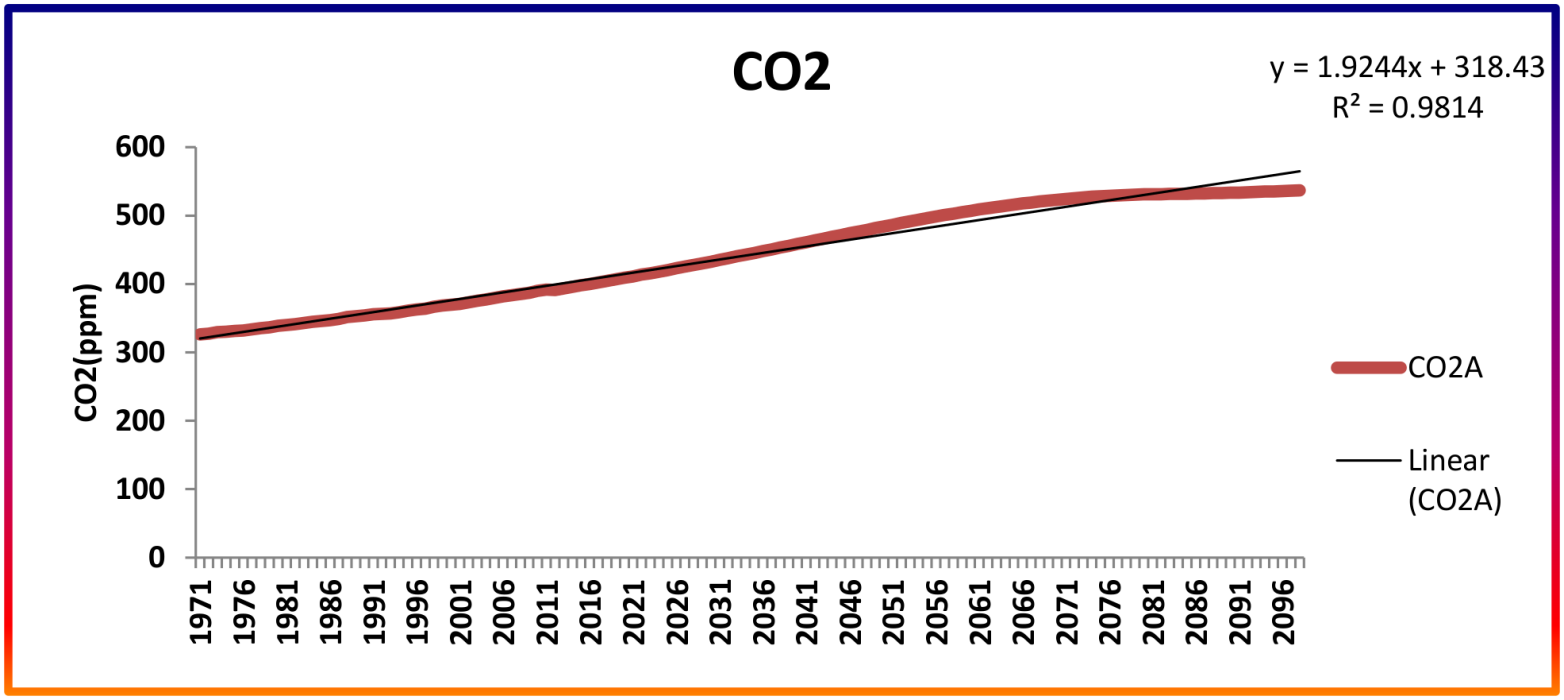
CO_2_ levels from RCP 4.5 of IPCC.

This study employed the dynamical downscaling approaches using the Regional Climate Model-RegCM 4.4 rc22 with HadGEM 2-ES lateral boundary conditions. The Regional Climate Model Version 4.4 (RegCM4.4) being developed by Abdus Salam International Centre for Theoretical Physics (ICTP), Italy, to simulate the future climate. It is an earth system model. For simulating the plausible future climate, the latest available emission trajectory (RCP 4.5) proposed by the AR5 report of IPCC was used (Fig 2).

The RCP 4.5, which is said to be in good coherence with the climate of the Indian subcontinent, was chosen for simulation [32]. This representative concentration pathway was chosen as it approximates the observed climatic conditions of an Indian scenario and that of the previous IPCC SRES A1B scenario. The period of study chosen was from 1971–2098 (128 years), considering 1970–2000 as the baseline, 2010–2040 as Near Century, 2041–2070 as Mid Century, and 2071–2098 as End Century. The 14 grid points covering the entire study area were extracted for analysis purposes.

#### Post processing of simulated output

**Fig 3:**
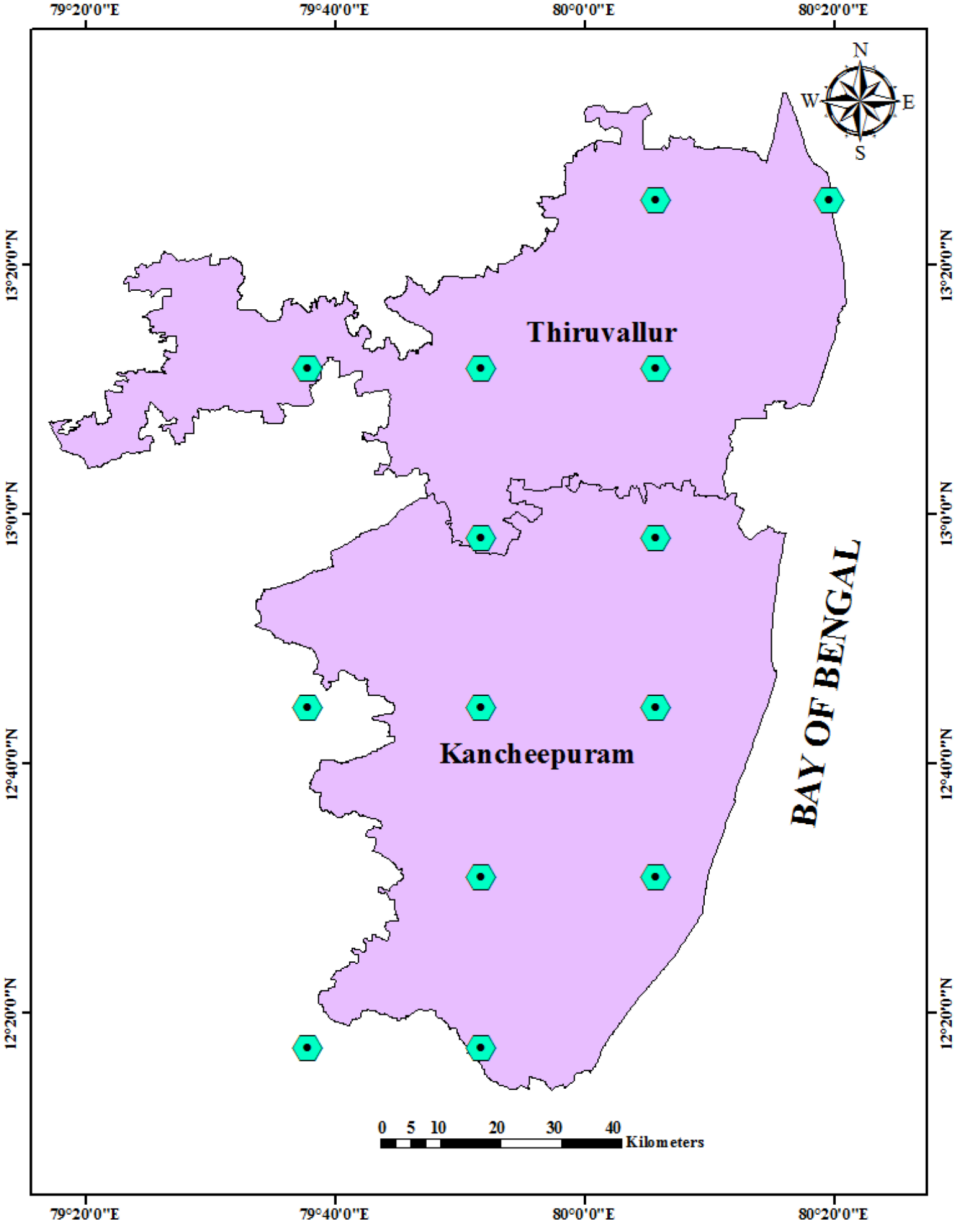
RegCM grid points covering the Chengalpet region.

The 14 grid points covering the entire study area were extracted for analysis purposes (Fig 3). The Arc GIS 10.2 was utilized to cull out the data pertaining to the study region. The simulation data of 88 years pertaining to Near Century (2010–2040), Mid Century (2041–2070), and End Century (2071–2098) climate scenarios, and the 30 year base line period (1970–2000) were analyzed. The major weather variables, including the daily maximum and minimum temperatures, the solar radiation, and the rainfall, were extracted from the outputs of the Regional Climate Models (RCMs). Since the HadGEM2 boundaries had a 360 day calendar, this was converted to the Gregorian calendar using methodology suggested by Minguez et al. [33]. From the daily outputs of Reg CM4.4, mean changes for the 30 year period and decadal estimations were made using the statistical software, SPSS.

#### Crop yield impact assessment

**Fig 4:**
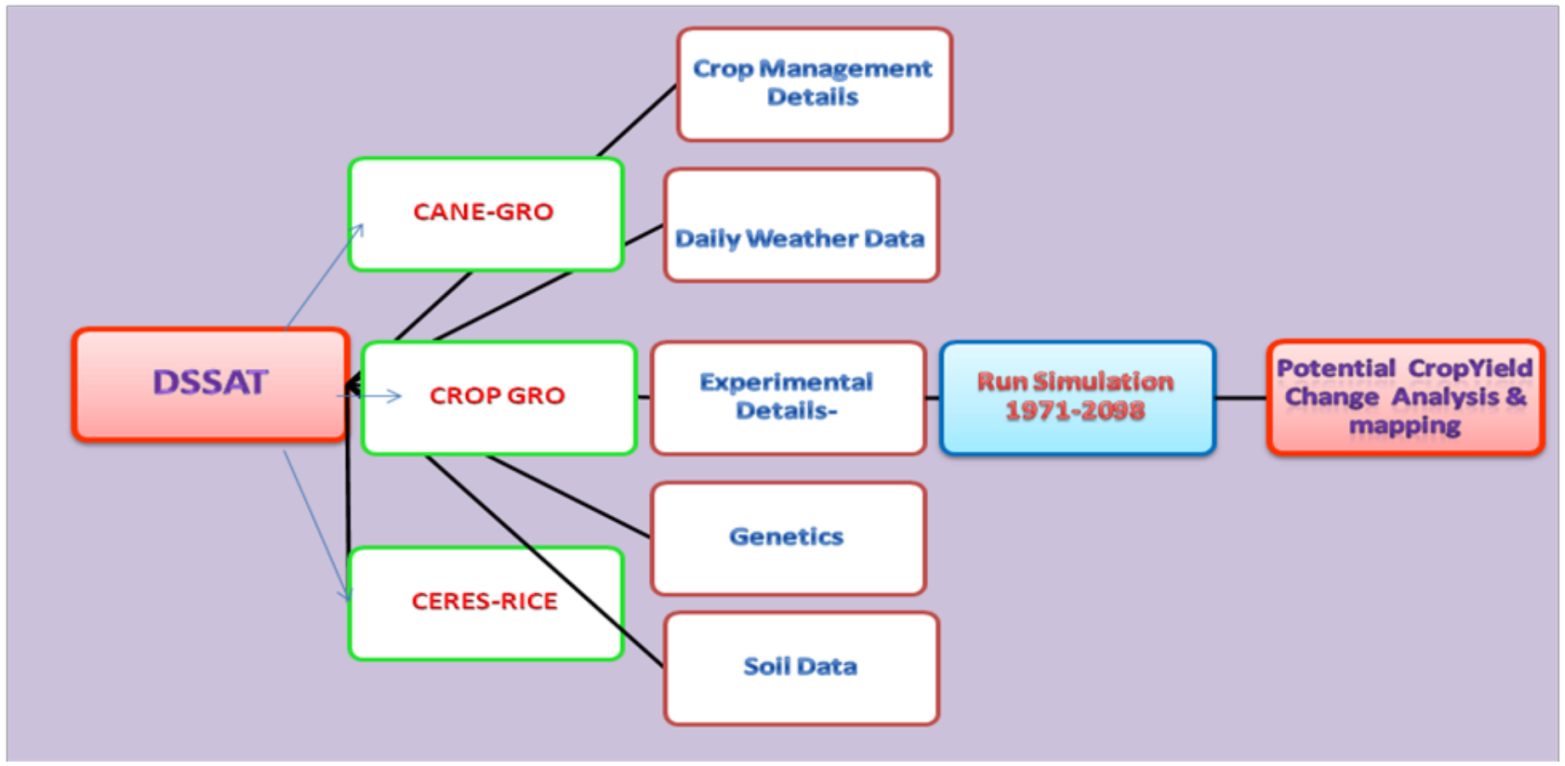
Impact assessment using DSSAT model.

The potential impact assessment on the yield of major crops was carried out using crop simulation models, which involve various processes. In this study, the simulation models, Decision Support System for Agrotechnology Transfer (DSSAT-V4.5) [34, 35], CERES-RICE under the cereal modules, CROP-GRO under the legume modules, and CANE-GRO under the sugar/energy modules, were chosen to simulate the future yield under varying climate scenarios (Fig 4). Information collected through the field work and the experimental results from the research centers of the Tamil Nadu Agriculture University were the major inputs for the simulations. Simulated daily weather files, soil files, and crop management files were created.

The CERES-Rice Plant Growth Module is process based and comes under the Grain Cereals-Rice compartment inside the DSSAT. This model emphasizes the effects of the weather, the management, and the soil properties on the crop performance [36]. The model was designed to predict the yield of crop varieties, the soil water regimes, and the nitrogen (N) level, considering alternative technologies and new crop sites as well [37–41]. Timsina and Humphreys [38] validated the output and mentioned that the CERES-Rice model has performed reasonably well in Asia and Australia. The performance of this model was evaluated using simulated and observed crop yield values at TNAU by various researchers.

The CROPGRO plant growth module is used widely to simulate the yields of legumes, including soybeans, peanuts, dry beans, chickpeas, cowpeas, velvet beans, and faba beans; vegetables, including tomatoes, bell peppers, cabbage, and green beans; forages, such as Bahia and brachiaria; and fiber crops like cotton. The effects of climate factors on the growth and the development of groundnuts have been simulated by the CROPGRO-peanuts in this study. It can be used to determine the impacts and adaptation of groundnuts to future climate change [42]. The major component of this model deals with the vegetative and the reproductive development, the carbon, the nitrogen, and the water balance [43].

The CANEGRO Plant Growth Module has been used widely to simulate the crop yield under changing climate and management conditions. This module is in the 'Sugar/Energy Crops—Sugarcane' compartment in the DSSAT model. The CANEGRO model developed by Inman-Bamber [44] could be a suitable tool to assist with this complex task of yield predictions. The CANEGRO model simulates the response of various sugar cane varieties under various agro-climatic conditions. It simulates the processes of water movement, such as runoff, gravitational flow, and root water uptake in a multi-layered soil profile. It also simulates canopy development, which is used to drive the energy balance of the crop by intercepting radiation for photosynthesis. Biomass is dynamically distributed among different components of the plant, including sucrose accumulated in the stalk, which is based on crop age, level of water stress, and temperature. Singels & Bezuidenhout [45] described a preliminary version (ver 5.1, 2002) of the model. The CANEGRO model has been validated for a wide range of agro-climatic conditions and generally simulates cane yield and sucrose yield satisfactorily [46, 47].

#### Post processing of the simulated output

The simulated outputs from the model was analyzed statistically and plotted using the Arc GIS tool. The daily values of the simulated outputs were converted into monthly values and then aggregated into 30 year mean periods starting from the baseline 1971–2000, and continuing through Near Century (2010–2040), Mid Century (2041–2070), and End Century (2071–2098). A spatial analysis was performed using the Arc GIS software on the relative yield changes of the major crops over the study area.

The relative yield change was calculated by the following equation:

$$\text{Relative Yield Change}=\left[\frac{\left(\text{Projected Yield}-\text{Baseline Yield}\right)}{\text{Projected Yield}}\right]\times 100$$

## Results and discussions

### Projected impacts on the crop yield

The slightest changes in the weather conditions may have a bearing on crop production, especially in an arid and semiarid tropical region like Tamil Nadu, India. This can become a prime factor influencing crop production. As the crops are highly susceptible to small changes in the microclimatic conditions, the estimates of future crop productivity at microscale would support appropriate actions in the future to curtail the potential impacts. Using the crop simulation models, the impacts on major crop yields were simulated and projected till the end of the 21st century by many researchers [45, 48–51].

The assessment of the impacts of future plausible climate change on crop yield was performed using DSSAT. The major crops in the study area, rice, groundnut, and sugarcane, performed in different ways in the emerging scenarios given by IPCC RCP 4.5. The future yield projections show that there will be decreases in the major crop yields selected for this study (Table 1).

**Table 1:** The mean projected crop yield changes under control and CO_2_ simulations.

Projected Yield	Year	Control	CO_2_ (538 ppm)
Rice	2010–2040	-8.8	-6.4
	2041–2070	-13.1	-4.7
	2071–2098	-18.7	-4.4
Groundnut	2010–2040	2.8	6.4
	2041–2070	-5.1	4.2
	2071–2098	-9.7	0.6
Sugar cane	2010–2040	-1.8	No Change
	2041–2070	-2.6	
	2071–2098	-2.8	

#### Impacts on rice crop yield

**Fig 5:**
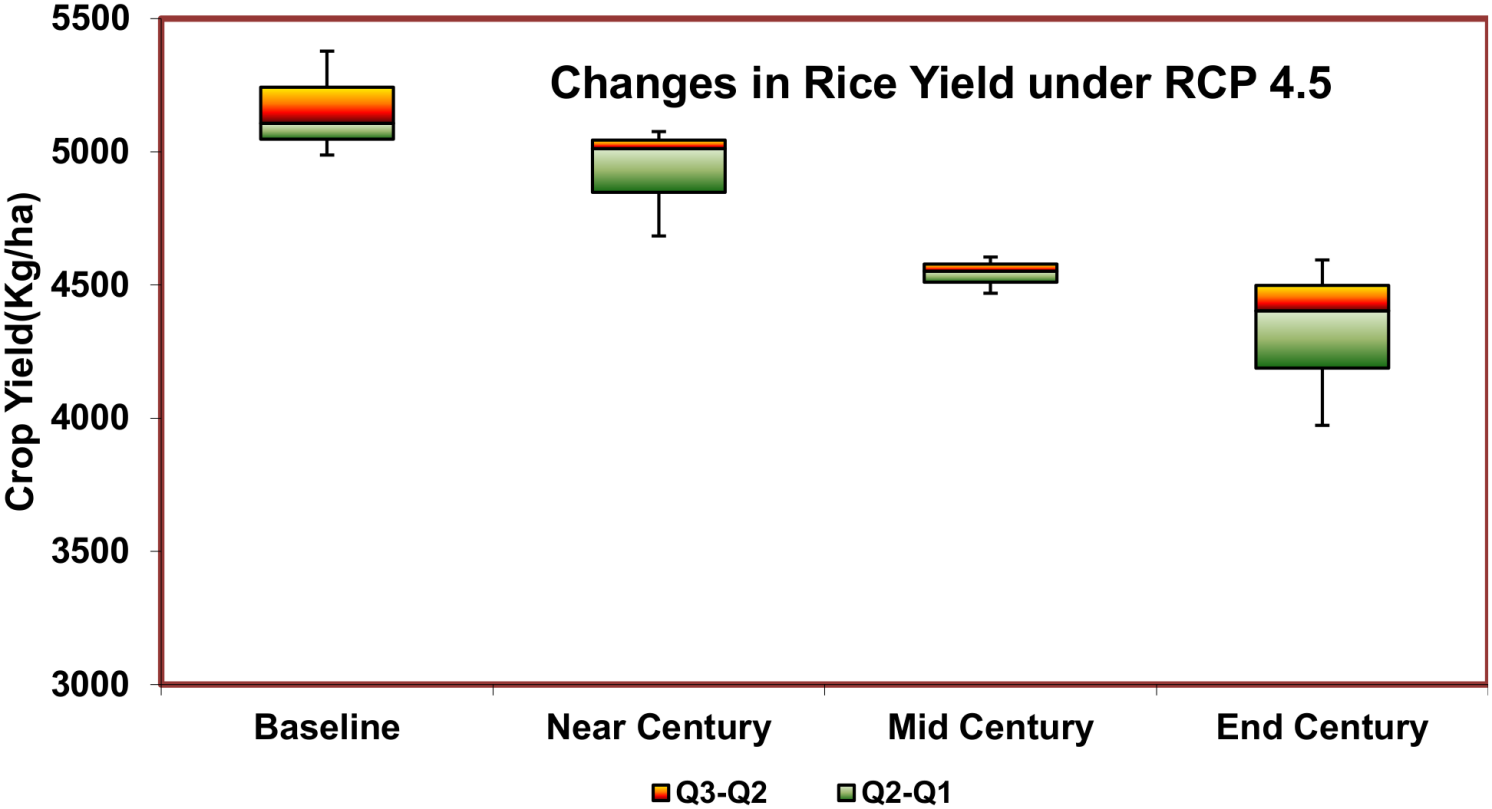
Projected avarage rice yield changes over near, mid and end century under RCP 4.5.

The assessment of the impacts of future plausible climate change on rice crop yield showed that the yield levels vary highly among the different rice growing areas within the study area as well as over the different time periods in the 21st century (Table 1). The ADT 43 rice variety, which is commonly grown in the study area, was used as the test variety; and the simulations were made with and without a CO_2_ fertilization effect. The yield projections using the CERES-Rice model showed that there will be a decrease in yield. The likely decline in yield for rice were 8.8%, 13.1%, and 18.73% under the control simulations for the time periods of Near, Mid, and End Century periods, respectively (Fig 5). In the case of the CO_2_ enriched scenario, the decline in the yield of rice was found to be 6.4%, 4.7%, and 4.4%, respectively.

A study carried out by Geethalakshmi et al. [39] projects a reduction in the yields of the same rice variety ADT 43 by 356 kg ha^–1^ decade^–1^ for PRECIS model output, whereas the decline was 217 kg ha^–1^ decade^-1^ for the RegCM3 output over the Cauvery Delta Zone simulated by the DSSAT without considering the CO_2_ fertilization. However, when the CO_2_ fertilization effect was considered, the PRECIS output showed a decreasing trend, whereas the RegCM3 projected an increased yield.

It has already been reported that an increase in CO_2_ concentration to 550 ppm could increase the yields of rice, wheat, legumes, and oilseeds by 10–20%. However, a one degree increase in temperature may reduce the yields of wheat, soybean, mustard, groundnut, and potato by 3–7% [13]. An increase in temperature, as expected, would result in decreased water availability [31], and its impacts on agriculture would be crucial for India.

**Fig 6:**
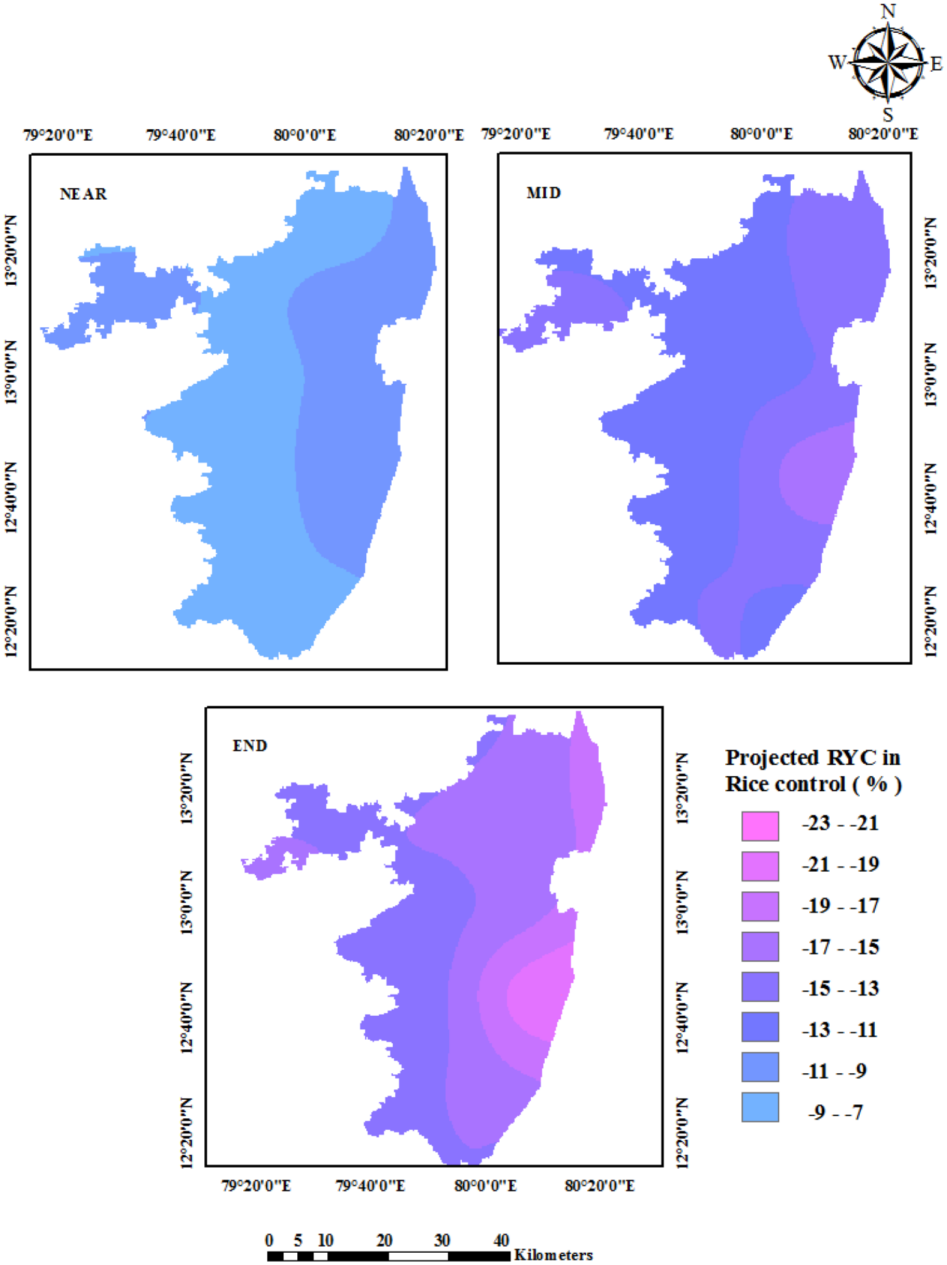
Spatial distribution of projected changes in rice crop yield under RCP 4.5.

The spatial distribution of the projected change in rice yield for the future had been assessed and represented in (Fig 6). As rice is highly sensitive to increases in ambient temperature variations, the simulation results produced by the CERES-Rice model showed that the crop yields under the elevated temperature conditions are likely to be affected severely. The crop yield reduction is likely to be least significant in the Near Century period as the yield reduction is projected to be 7 to 10%. The reduction is projected to be 11 to 15% in the Mid Century and in the range of 13 to 21% by the End Century period.

The blocks of Tirukalikundram, Lathur, Tiruporur, Minjur, Sholavaram, R.K. Pet, Ellapuram, etc. are likely to be affected more significantly. The rice cultivation needs to be done with care, and new varieties, which are heat and salinity tolerant, are needed.

#### Impacts on groundnut crop yield

**Fig 7:**
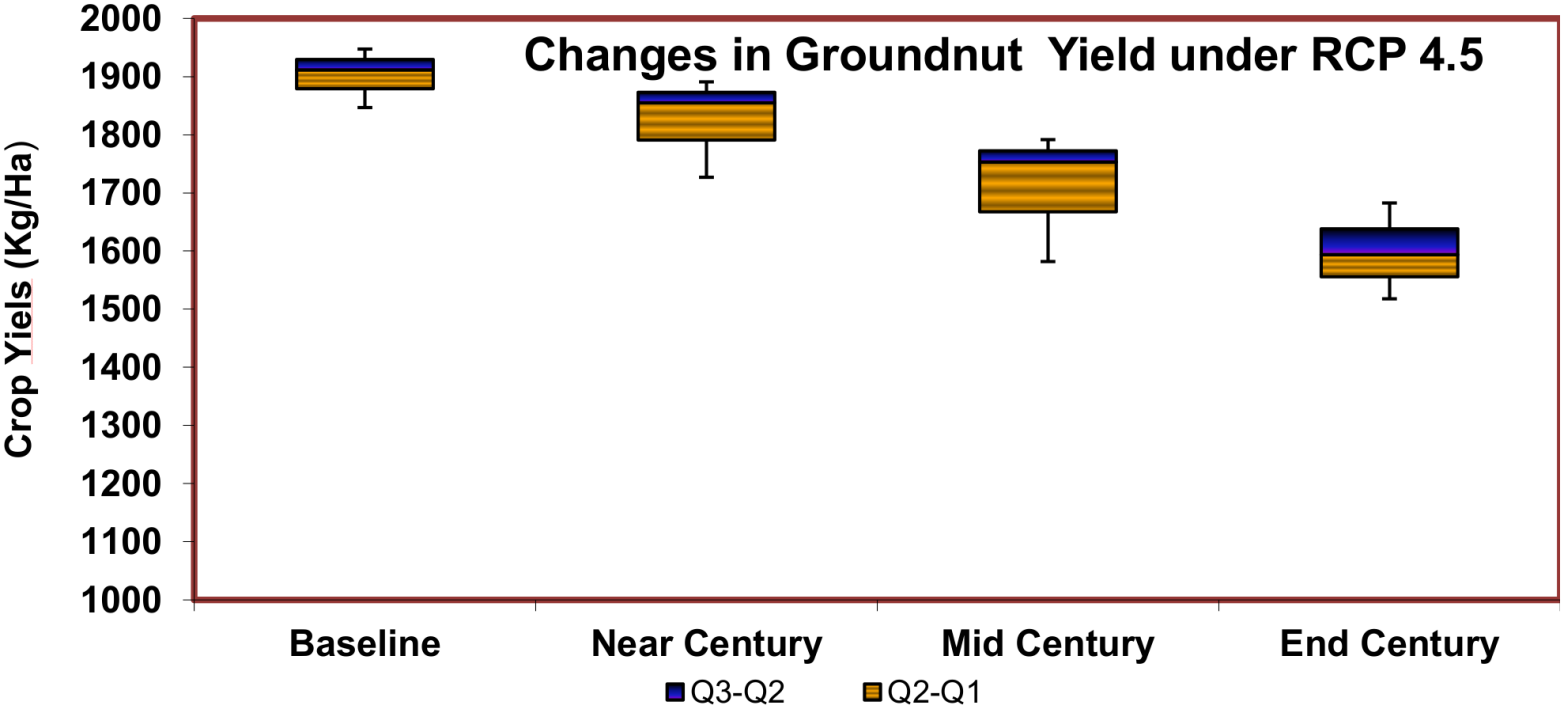
Projected avarage groundnut yield changes over near, mid and end century period under RCP 4.5.

The relative yield change for groundnut by the End Century period was negative in the control simulations. The yield is likely to be reduced by 5.1 and 9.6% for the Mid and End century as shown in Fig 7. However, under a CO_2_-enriched scenario, there is a yield increase until the 2060s, after which the yields begin to decrease.

**Fig 8:**
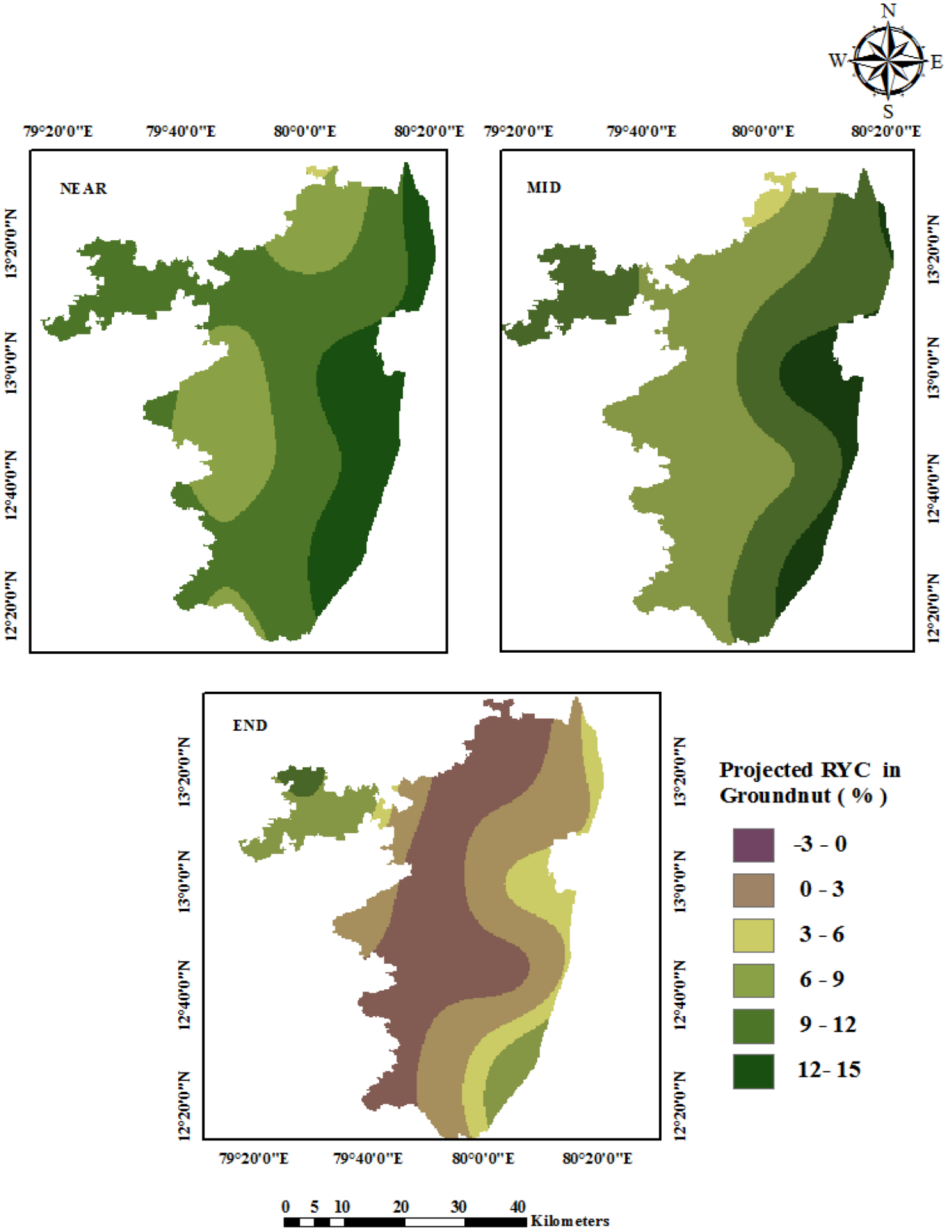
Spatial distribution of projected crop yield changes in groundnut under RCP 4.5.

The spatial distribution of projected crop yield changes for groundnut is shown in Fig 8. The relative yield changes in the groundnut crop shows a positive change for the Near and Mid Century simulations. During the End Century period, due to large changes in the temperature in the daytime as well as in the night time, the groundnut yield may tend to decline or fall by -3%. As far as relative yield change is concerned, the northwestern parts of Acharapakkam, Uttiramerur, Wallajabad, Kadambattur, Poondi, Gummidipoondi, etc., are likely to be affected badly. The other part of the study area will see a slight increase in groundnut yield under the changing scenarios of climate change.

#### Impacts on sugarcane crop yield

The relative mean yield change for sugarcane showed only a slightest decrease of 1.8%, 2.6% and 2.8% for the Near, Mid, and End Century periods. The impact of the CO_2_ fertilization (+520 ppm) was indicated until the Mid Century period.

**Fig 9:**
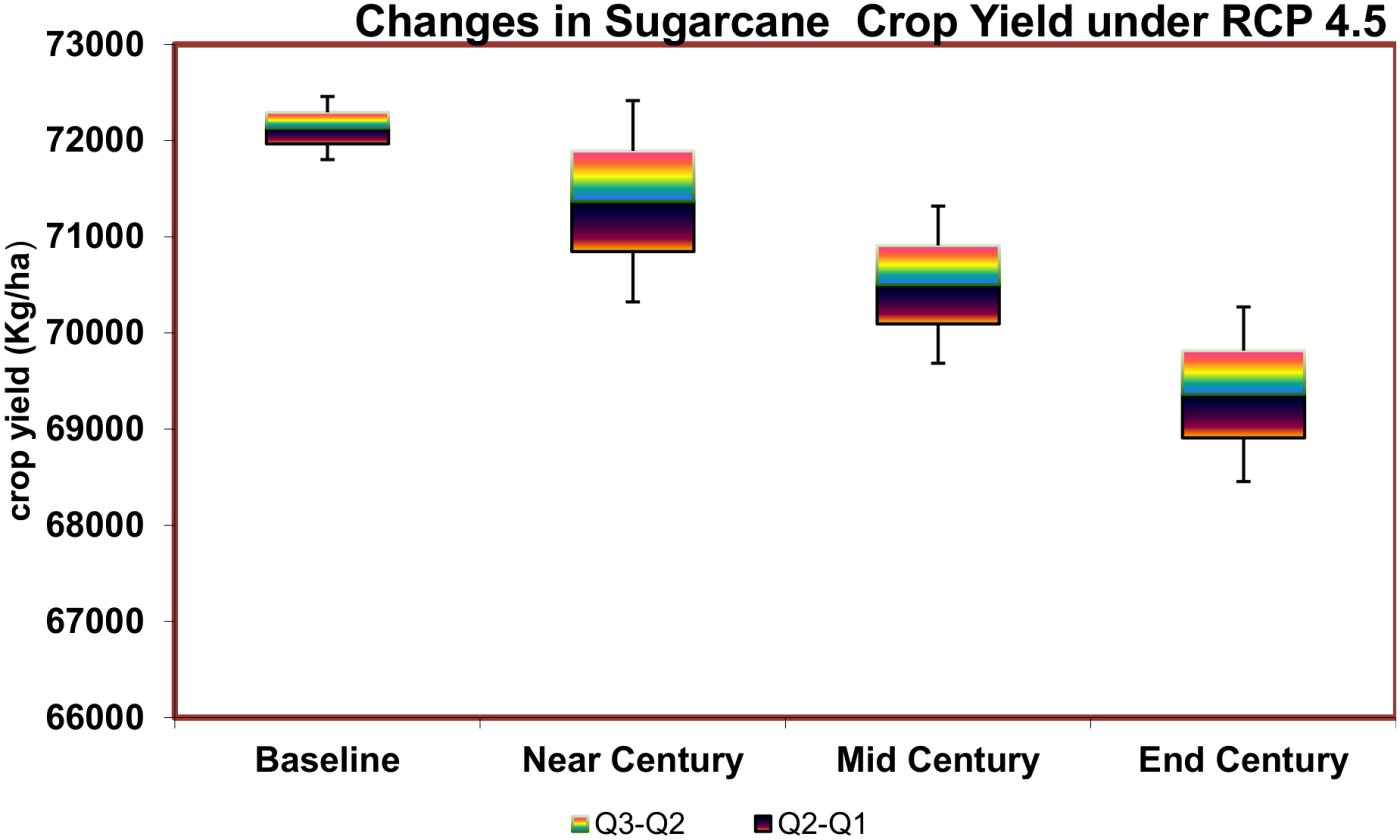
Projected avarage groundnut yield changes over near, mid and end century period under RCP 4.5.

The projected yield of sugarcane during the End Century may be reduced by 3 to 1% (Fig 9). The relative yield change shows that the yield decline would be significant in the majority of the area in the interior of the study area. In the Near and Mid Century periods, there is likely to be a slight yield increase noted near the northeastern coastal areas. However, during the End Century period, little variation was noted even under the elevated CO_2_ conditions. This may be because sugarcane is a C_4_ crop.

**Fig 10:**
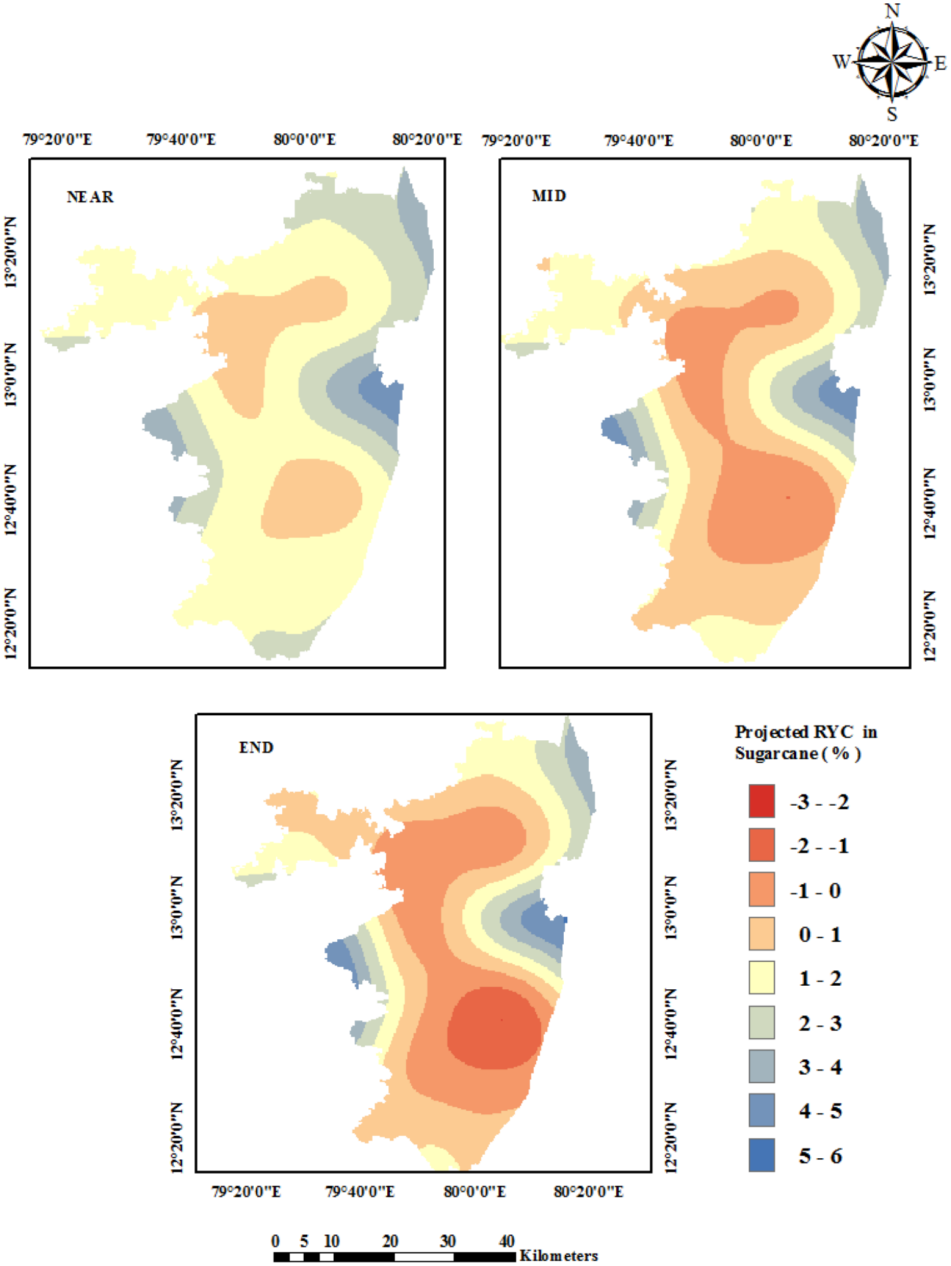
Spatial distribution of projected crop yield changes in sugarcane under RCP4.5.

Under the RCP 4.5, in the scenario of a mean temperature rise of 2.4°C, there is not much decrease in the sugarcane yield during the End Century compared to the other two crops. The crop productivity in the areas of Thirukalikundram, Lathur, Uthiramerur, Wallajabad, Thiruvalangadu, Tiruttani, Thiruvallur, and Poondi blocks are likely to be affected more in the Mid and End Century periods (Fig 10).

Generally, in many Asian countries, rice provides up to 80% of the energy intake of the poor. Any negative impacts on the quality and quantity of rice yield would affect the local food security [51–53]. It is very important to focus on the performance of crops under the changing scenarios for climate, as it is very central to the wellbeing of humans. Hasanuzzaman [14] has reported that physiological responses in plants may occur at short and long time scales as a result of unusual surface warming. This present study endeavored to understand how the plausible changes in the future weather conditions under RCP4.5 would be reflected in the changes in the crop production.

In this study, under the enhanced emission pathway under RCP 4.5, the CO_2_ fertilization (+520 ppm) effect was seen until the Mid Century period, as there were some peaks in crop yield. After that threshold level, the yield of rice and groundnut was seen to decline, probably due to greater warming. By evaluating all three crops, it is possible that there will be a definite decline in yield under the controlled conditions (without changing the level of CO_2_,) as discussed earlier. Under the CO_2_-enriched condition, the rice and groundnut crops responded better. However, sugarcane did not react to the CO_2_ enrichment. This might be due to the difference in their CO_2_ pathways. A successful adaptation at the local level requires a proper plan with short and long term perspectives that can be addressed using multiple pathways [54]. A variety of options can be put forth while planning for adaptations, including developing salt, heat and drought tolerant crops, adjusting the timing of farm operations, changing crop varieties and patterns, improving water use efficiency, and developing farm risk insurance [55, 56].

Changes in global temperature are accompanied by certain changes in precipitation patterns, enhancing the risk of climate induced extreme events like floods [57], droughts [58], tropical cyclones [59], and frequent heat waves. In an agrarian economy like that of India, the agricultural production sector still provides 50% of the employment opportunities and contributes up to 14% of the total gross domestic product (GDP) of the country. About 43% of the total geographic area is being used for agricultural purposes in the country, and about 60% of the net sown area is still rainfed [60]. Agriculture in India is highly sensitive to climate change impacts [61–65]. For example, factors, such as a high dependence on monsoons, small land holdings, and excessive reliance on fertilizers, contribute to the existing vulnerability of the Indian agricultural sector.

## Conclusions

Generating plausible crop yield change scenarios is possible with the help of regional climate and crop simulation models. The DSSAT crop simulation model is a very constructive tool for making important decisions for future agricultural operations. Major negative impacts with respect to changes in climate and crop yield are noticed for the rice crop, which is a major food crop in India. A more detailed study using a high resolution climate model at the village level would provide promising outcomes for micro-level adaptation assessments. Furthermore, this study has revealed that without timely adaptation, the food security and livelihood security would be at it risk using the climate change scenarios as described in RCP4.5. It is necessary to have a synergistic adaptation assessment and implementation plan that combines field level and scientific inputs (community based adaptations), but that also enhances the inherent capacity of the crops (ecosystem based adaptations) to withstand any future changes in climate. This study has also provided a systematic approach to explicitly identify vulnerable areas with respect to diminishing crop yields, where regional planners and policy makers can build on existing adaptation decision-making by utilizing an interdisciplinary approach in the context of global change scenarios.

The results of the impact assessment show the areas that may require extra focus in crop production in the semiarid agro-ecological zones of South India. Micro-level analysis with high resolution climate details would be highly beneficial for better adaptation at the village level. These areas exhibit the highest degree of negative variation in crop yield, with conditions of high vulnerability expected in the later part of the 21st century. The study emphasized the priority for implementing adaptive management actions in the identified areas.

## Supporting information

S1 File (ZIP)
