# Supplementary figures and images for: Spatiotemporal analysis of projected impacts of climate change on the major C3 and C4 crop yield under representative concentration pathway 4.5: Insight from the coasts of Tamil Nadu, South India
Source: PLoS One. 2017 Jul 28;12(7):e0180706. doi: 10.1371/journal.pone.0180706 (PMC5533328; doi:10.1371/journal.pone.0180706)

## Slide 1
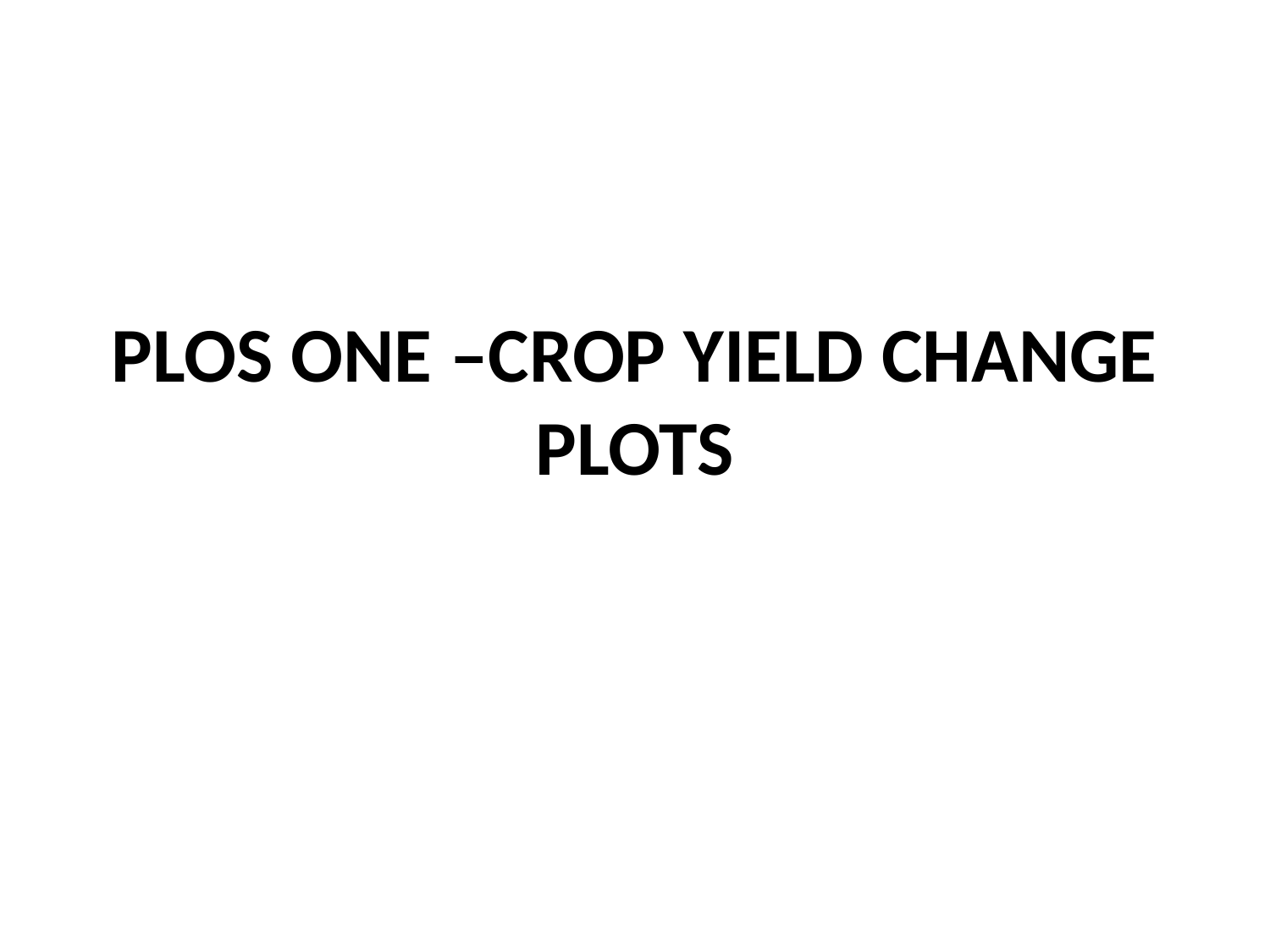

## Slide 2
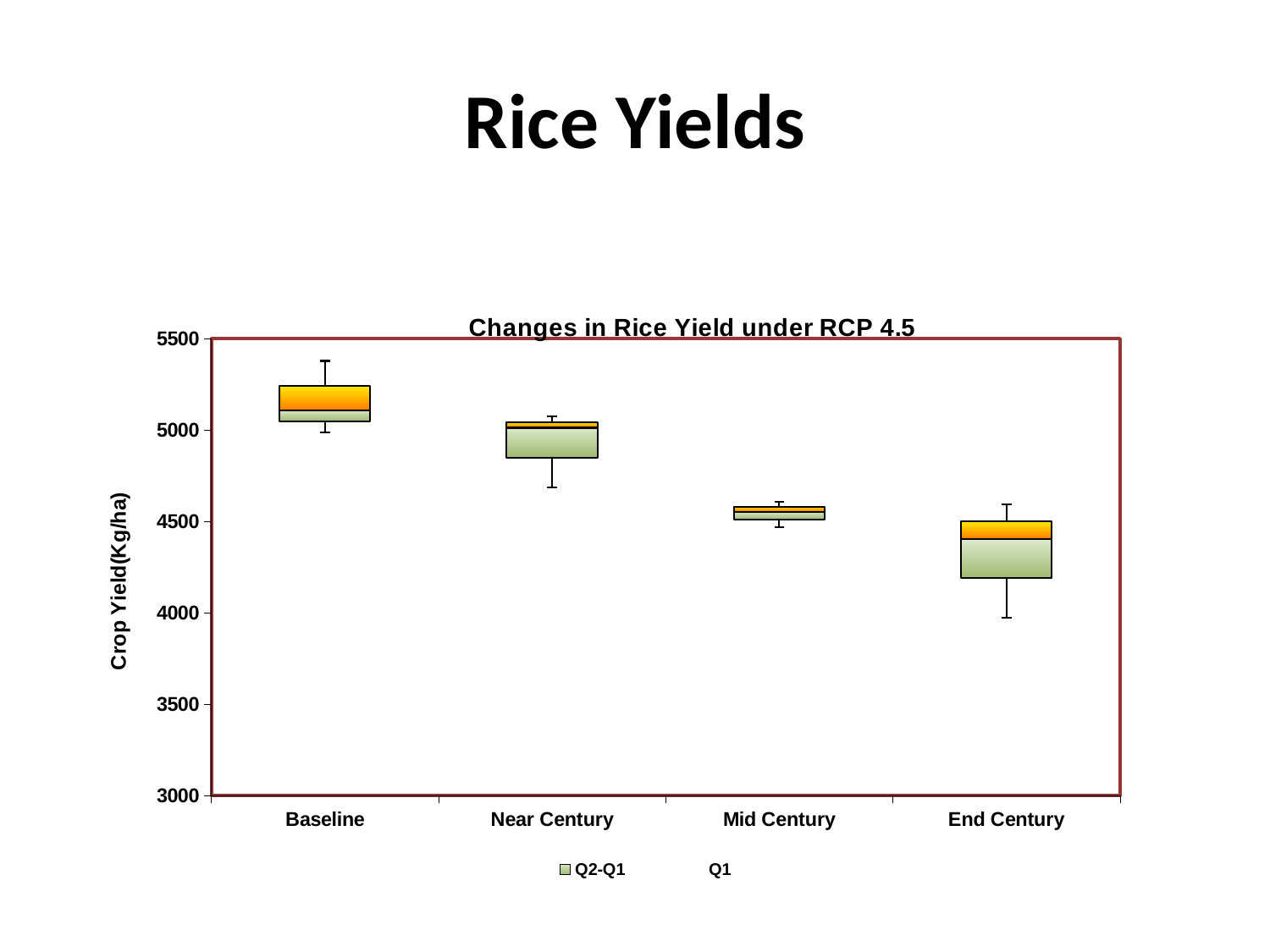

## Slide 3
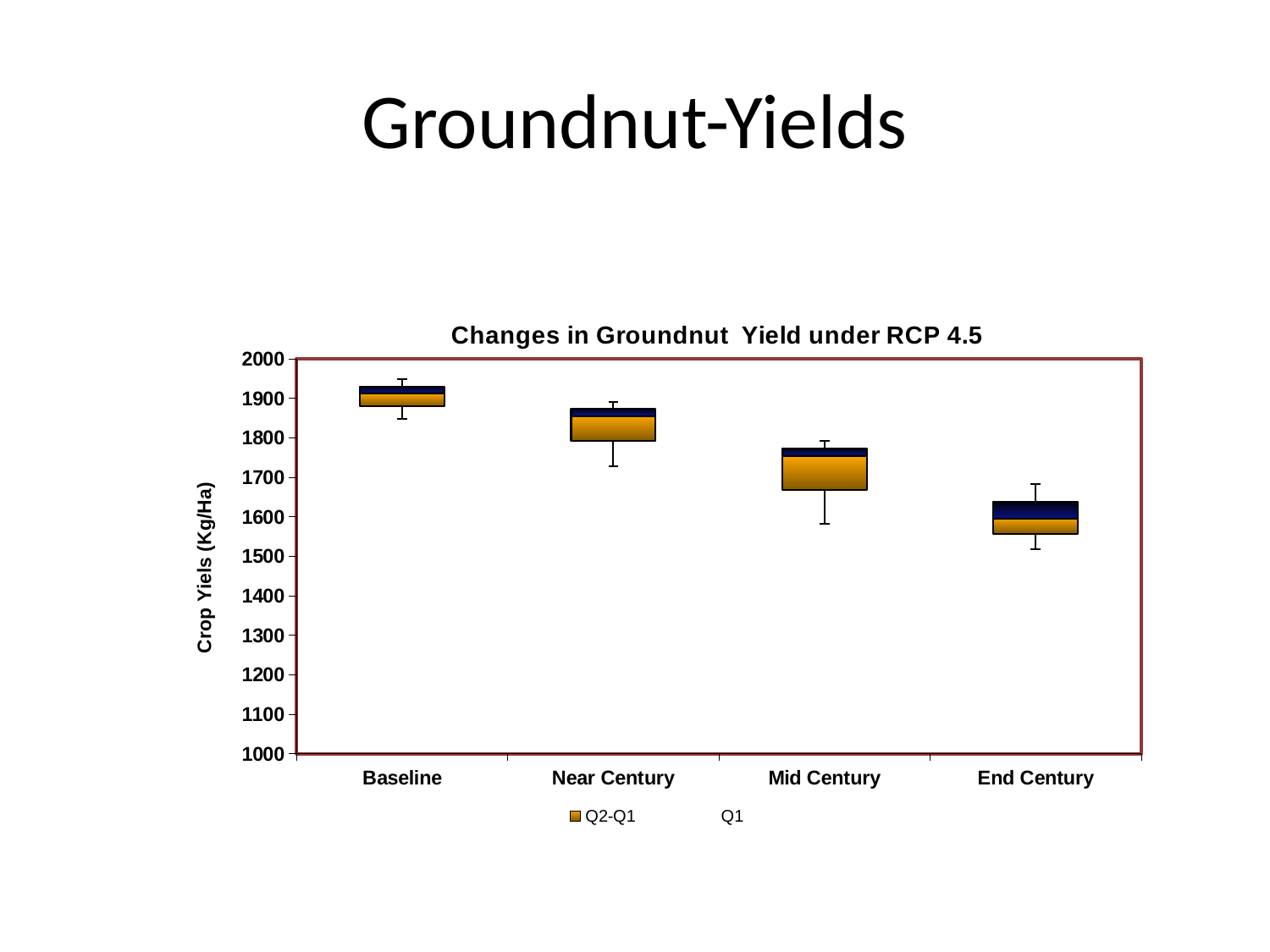

## Slide 4
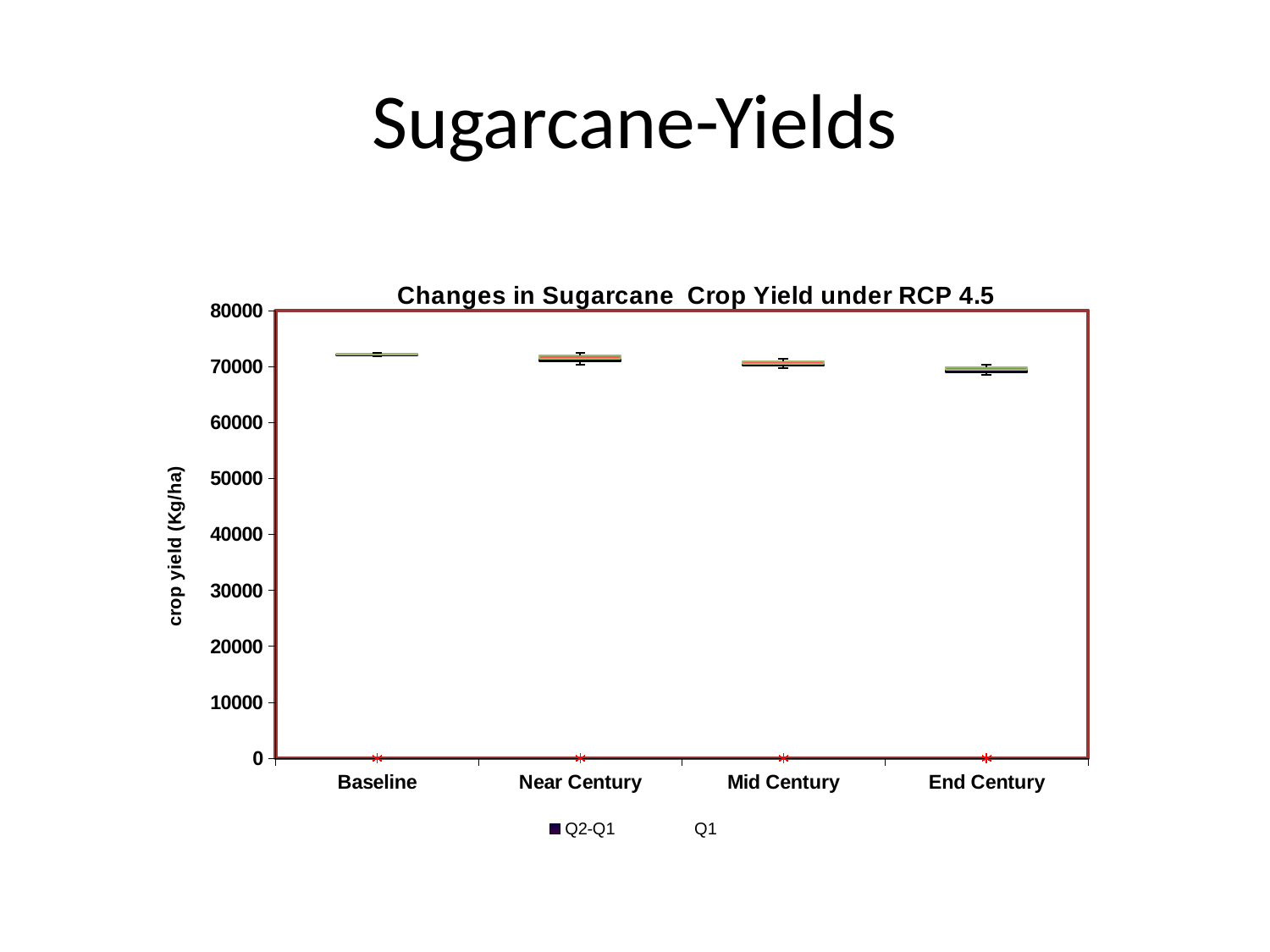

Supplement: S1 File — (ZIP) [file pone.0180706.s001.zip › renamed_837d6.pptx]
